# Navigating the Pandemic: An Exploration of Medical Practitioners’ Preparedness, Concerns, Adaptations, and Practices During the COVID-19 Epidemic in India

**DOI:** 10.7759/cureus.48677

**Published:** 2023-11-12

**Authors:** Anjali Modi, Kishor Jadhav, Krupal J Joshi, A M Kadri, Ashish K Naik

**Affiliations:** 1 Community and Family Medicine, All India Institute of Medical Sciences, Rajkot, Rajkot, IND; 2 Epidemiology and Public Health, Ratnagiri District Health Department, Dapoli, IND; 3 Community Medicine, State Health System Resource Center, Ahmedabad, IND; 4 Epidemiology and Public Health, Surat Municipal Corporation, Surat, IND

**Keywords:** sars-cov-2 (severe acute respiratory syndrome coronavirus -2), coronavirus disease 2019, healthcare providers, ivermectin, covid vaccine, challenges, treatment practices, covid-19 management, private sector, doctors

## Abstract

Introduction

The coronavirus disease 2019 (COVID-19) pandemic, caused by the severe acute respiratory syndrome coronavirus 2 (SARS-CoV-2) virus, has had profound health and societal impacts, and healthcare providers from diverse backgrounds had to continuously adapt and update to manage patient care, prevent morbidity-mortality, and minimize transmission of the infection.

Methodology

A cross-sectional survey was conducted among 218 doctors in western India. A structured questionnaire was used to gather data on demographic characteristics, patient consultations, infection prevention practices, COVID-19 diagnosis, management, vaccination attitudes, and healthcare program disruptions. Multistage probability sampling was undertaken to select 161 (64%) private and 57 (26%) public sector doctors from the list of clinics and hospitals reporting COVID-19 cases in the urban municipal corporation area of South Gujarat. Private sector doctors were contacted through the network of public administrative staff and caregivers of their area. They were provided the choice of date, time, and mode (telephonically, face to face, or online) of interview. Descriptive measures of central tendency and variation were calculated. Inferential statistics was applied to test the significance of the difference between sub-groups. For ratio and interval variables, t-test (for two groups) and ANOVA (for more than two groups) were applied while for nominal and ordinal variables, chi-square and appropriate tests were applied.

Results

The mean age of the 218 doctors included in the study was 43.6 ± 11.1 years while the mean duration of practice was 16.9 ±10.8 years. During the pandemic, patients’ consultation frequencies decreased at the clinics while telephonic and residential consultancies increased, which was statistically significant (P=0.000). Social distancing (n= 187; 85%), isolation (n=157; 72%), and consultation reduction (n=65; 30%) were adopted by doctors. Both public and private doctors preferred government-recognized COVID-19 centers for testing (n=167; 76.7%) and reverse transcriptase-polymerase chain reaction (RT-PCR) as the standard diagnostic test (n=196; 90%). A combination of antipyretics, favipiravir, and antibiotics was used to manage symptomatic cases. Concerns and emotional stress for personal and family safety were prominent among this group of frontline medical doctors (94%). Delivery of healthcare programs for chronic conditions like hypertension and tuberculosis was negatively affected (n=102; 47%). Despite these challenges, doctors managed cases and advised vaccination to control the pandemic.

Conclusion

This study among over 200 qualified medical practitioners during the pandemic attempts to fill gaps in COVID-19 management, prevention, and safety measures. To the best of our knowledge, this is one of the few studies providing genuine insights into the practice of private doctors with a large sample size. Findings show the established treatment, prophylaxis, and vaccination protocols among private and public practitioners. It highlights the need for adaptable healthcare strategies and collaboration between public and private sectors for managing future global health emergencies.

## Introduction

Coronavirus disease 2019 (COVID-19) is caused by a novel human coronavirus, severe acute respiratory syndrome-corona virus (SARS-COV-2), an enveloped single-stranded RNA virus, previously known as 2019-nCov [1]. As of January 2022, the World Health Organization (WHO) reported approximately 96,012,792 confirmed cases, with 2,075,870 deaths globally, and 10,625,428 confirmed cases and 153,302 deaths in India, giving the disease a status of pandemic and global emergency [2-4]. The COVID-19 pandemic created a humanitarian crisis with high levels of infection, morbidity, and mortality among healthcare personnel [5-7].

COVID-19 infection is mild in 95% of cases but may require hospitalization in the remaining patients who develop severe acute respiratory syndrome (SARS) or acute respiratory distress syndrome (ARDS). Due to the contagious nature of the epidemic, thousands of people fell sick at the same time requiring home isolation and quarantine [4,7], and the ones who developed ARDS required intensive care and hospitalization. Even the most developed and advanced healthcare systems of the world were not prepared for this challenge [5,8].

This unique nature of the COVID-19 pandemic and the initial absence of effective drugs and vaccines generated fear, stigma, and stress among doctors [9,10]. India has nearly twice as many private hospitals as public ones and 70% of the population prefer to seek healthcare from private sector hospitals [8,11]. For effective management of the situation, it was required that both public and private doctors contribute to COVID-19 care [6,8,12].

There is a dearth of published studies on COVID-19 management strategies, challenges, and evolving health practices of doctors that include both public and private sectors in India [3,10,13,14]. The present study plans to capture and document the required information to fill this gap. The findings from this study are envisaged to inform and frame future strategies for ensuring the preparedness of the healthcare system during global health emergencies and pandemics.

## Materials and methods

Study settings and design

This was a cross-sectional study conducted among the private and public sector doctors in an urban municipal corporation area of South Gujarat, India, catering to a population of one million in western India. A pre-designed, semi-structured questionnaire was prepared to focus on study objectives and available literature to study the medical practices, patterns, and influencing factors during the COVID-19 pandemic. The study was approved by Human Research and Ethics Committee of the Institutional Review Board of Government Medical College, Surat, Gujarat, India (vide letter number GMCS/STU/ETHICS/Approval/764/21, dated January 27, 2023). The personal identifiers were removed from datasheets to maintain the confidentiality of the study participants.

Sampling frame and sample size

Multistage systematic random sampling was planned for this study. The Surat Municipal Corporation (SMC) online surveillance system, SMC App (Jaimini Software, Surat, Gujarat, India) shows 1500-1800 private doctors with fever clinics. Approximately 500 private practitioners from the total surveillance network reported the majority of acute respiratory infections (ARI) and severe respiratory infections (SARI) cases. Out of these, it has been estimated that 80% or more were of patients with COVID-19-like symptoms.

**Figure 1:**
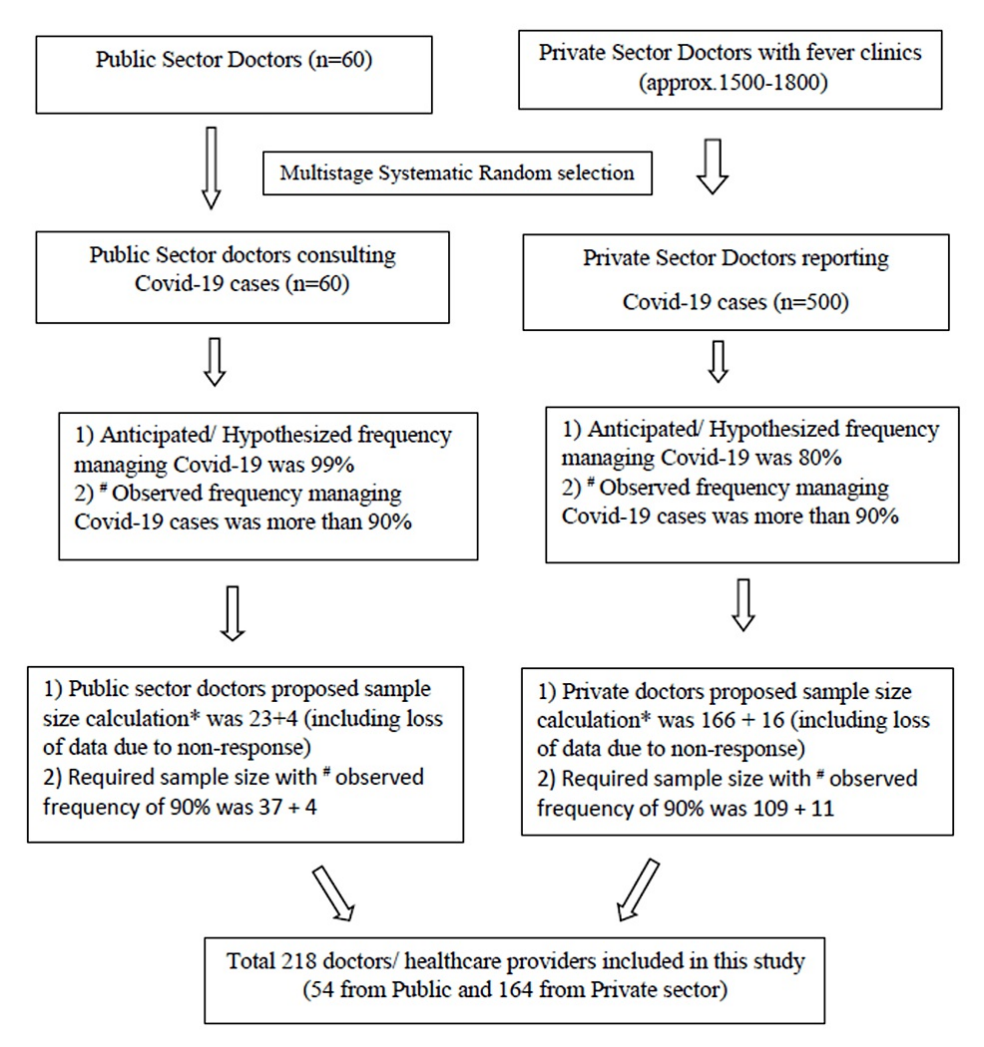
Flow diagram of the study design, sample, and methods Sample size calculations were done with OpenEpi online software; COVID-19 pandemic and private sector involvement were observed during the study and calculation of sample size was done based on actual statistics. COVID-19: coronavirus disease 2019

OpenEpi online sample size calculator (www.OpenEpi.com) was used to calculate the sample size; entering population size for private sector doctors, n=500, and the proportion of variable of interest as 80%, the sample size proposed was 166. During the study, it was observed that more than 95% of private-sector doctors were consulted for COVID-19. Taking the observed frequency of 90%, the sample size was 109. Adding the 10% for non-response and loss to data (n=11), we get the final sample size of 120 for private doctors (Figure 1). In the public sector, all urban health center doctors (n=60) consulted COVID-19 cases. Using multistage sampling methods in this group of public doctors and 90% as the frequency of providing COVID-19 consultation, the final sample size was 41 for the government sector.

Recruitment

The list of doctors reporting COVID-19 patients to the health management system was obtained from responsible officers to select the study participants. With the help of the participant information sheet (PIS), the study was explained, and written informed consent was taken on the participant informed consent form (PICF). The participants were provided the choice of a suitable date, time, and mode (telephonically, face to face, or online) for the interview. Daily monitoring was done for the completion and quality of responses. Private sector doctors were contacted through a network of public administrative staff and caregivers of their area. To ensure the quality of data, all interviews were directly observed and study participants were encouraged to ask questions related to proforma.

Data analysis

The data was entered in Google Forms (Google LLC, Mountain View, California, United States) during face-to-face or telephonic interviews. Descriptive measures of central tendency and variation were calculated and described with the help of tables, charts, and box-and-whisker plots. Inferential statistics was applied to test the significance of the difference between sub-groups. For ratio and interval variables, t-test (for two groups) and ANOVA (for more than two groups) were applied while for nominal and ordinal variables, a chi-square test was applied. Microsoft Office Word and Excel (Microsoft Corporation, Redmond, Washington, United States) and IBM SPSS Statistics for Windows, Version 26.0 (Released 2019; IBM Corp., Armonk, New York, United States) were used for data analysis.

## Results

Socio-demographic characteristics

**Figure 2:**
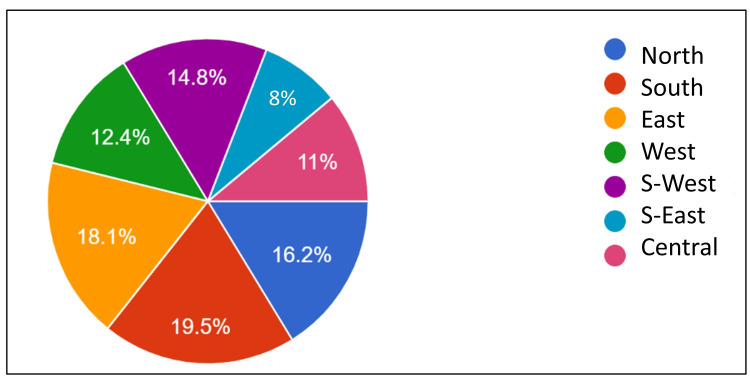
Proportion of study participants from each zone

**Figure 3:**
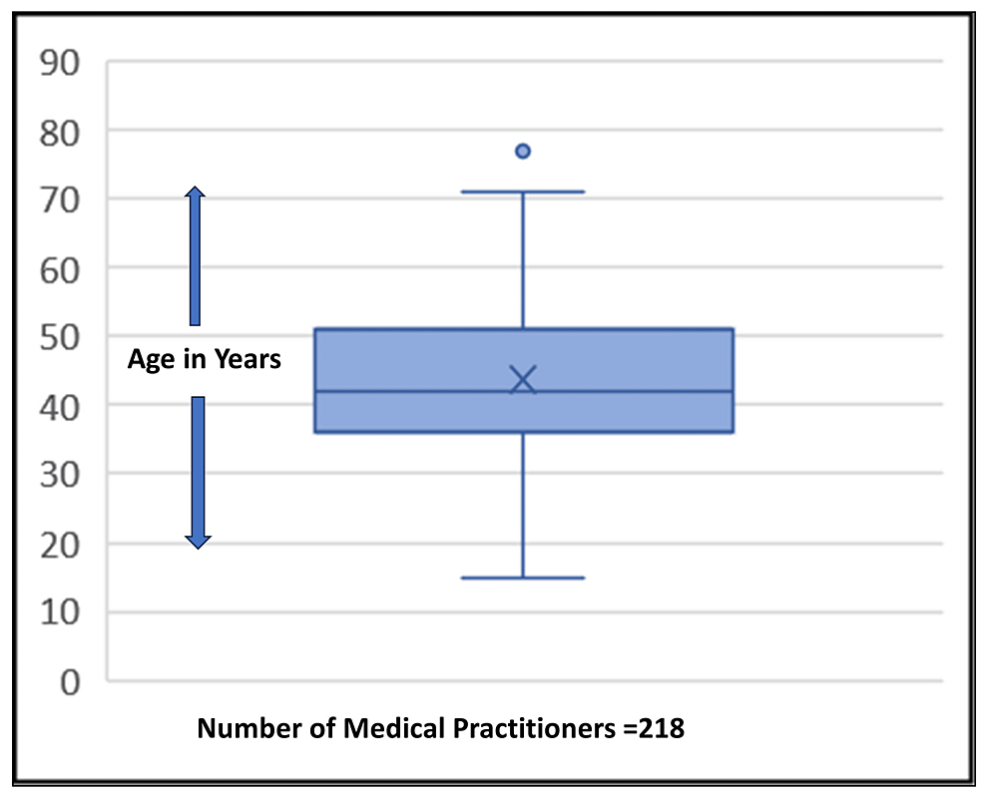
Box-and-whisker plot of the age distribution, numerical values The box shows the distribution of 50% of doctors (interquartile range) while the outside lines show the minimum and maximum ages. "X" in the box shows the position of measure of central tendency, median. The single dot on top shows the outlier and maximum age of 77 years.

**Figure 4:**
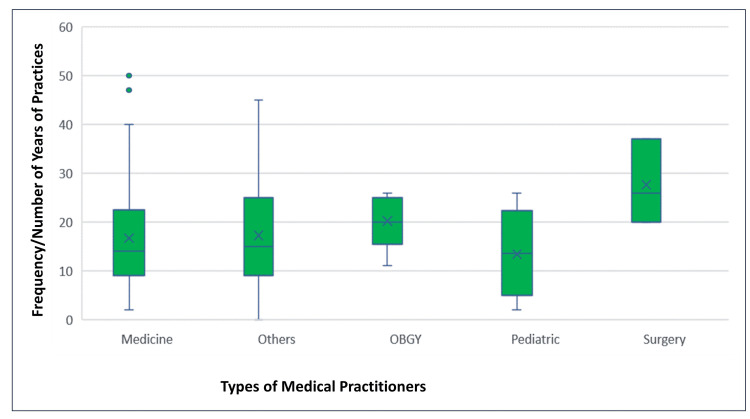
Box-plot chart showing the duration of doctors’ practice in years The box shows the 50% values while the last point of whiskers show the minimum and maximum years of practice. The two dots outside first box show the outliers/extremes of years of practice (47 and 51 years, respectively) compared to other values.

The present study involved 218 urban doctors practicing in seven administrative zones of the city (Figure 2). The mean age was 43.6 + 11.1 years (mean ± SD), the median was 42 years, and the modal age was 37 years for this group of doctors/healthcare providers (Figure 3). In this study, 57 (26%) doctors worked in public clinics and hospitals while the remaining 161 (74%) provided their services in privately established clinics or hospitals. The mean duration of practice was 16.9 ±10.8 years. Nine doctors had a practice of more than 40 years (Figure 4). Half of the doctors (n=113; 51.5%) were general practitioners: 47 (41%) from the government sector and 66 (58%) from the private sector. 

Healthcare utilization and patient consultation

It was noteworthy that more patients consulted the doctors at their clinic in one week before (mean=214) compared to during (mean=165) the COVID-19 pandemic. The difference between the average number of cases (mean, median, and mode) who required consultation with doctors in their clinic, homes, or phone at different stages of the COVID-19 pandemic was found statistically significant by paired t-test and probability values (P=0.000) (Table 1).

**Table 1:** Comparison of average patient consultation in one week before and during the COVID-19 pandemic COVID-19: coronavirus disease 2019

Patient consultations by doctors (n=218)	Mean	Standard deviation	Mean standard error	Correlations (p-value of significance)	t-test (p-value of significance)
At clinic, Before COVID-19 pandemic	214.56	209.71	14.24	0.602 (0.000)	3.196 (0.000)
At clinic, During COVID-19 pandemic	165.48	203.76	13.83		
At home, Before COVID-19 pandemic	4.94	21.04	1.43	0.757 (0.000)	-2.819 (0.005)
At home, After COVID-19 pandemic	8.44	27.61	1.87		
Over phone, Before COVID-19 pandemic	9.12	24.09	1.65	0.783 (0.000)	-4.58 (0.000)
Over phone, after COVID-19 pandemic	15.56	33.34	2.28		

Infection prevention practices

Standard precautions (89%) was the method of choice to prevent transmission of infection followed by social distancing (n=185, 85%) and reducing the number of patients and hours of consultation during the pandemic (chi-square value 10.6 and P<0.001) (Table 2).

**Table 2:** Comparison of infection prevention practices before and during (after) the COVID-19 pandemic ^*^t-test values significant with p-value < 0.05 COVID-19: coronavirus disease 2019

Infection prevention practices followed by doctors (n=218)	Before COVID-19 pandemic, n (%)	During and after COVID-19 pandemic, n (%)
Standard precaution	188 (85.8)	196 (89.4)
Social distancing*	118 (53.8)	187 (85.3)
Closing hospital	11 (5.0)	19 (8.6)
Reducing number of patients *	35 (15.9)	65 (29.6)
Referral	115 (52.5)	131 (59.8)
Others	39 (17.8)	50 (22.8)

COVID-19 management and vaccination

**Figure 5:**
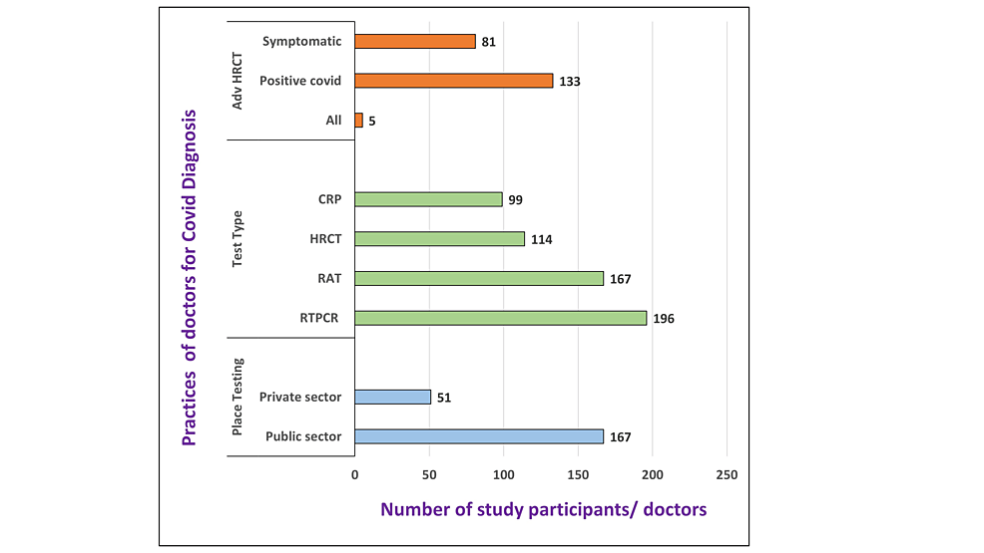
COVID-19 diagnosis practices among medical professionals (N=218) RT-PCR: real-time reverse transcriptase-polymerase chain reaction; CRP: C-reactive protein; RAT: rapid antigen test; HRCT: high-resolution computed tomography; Adv: advise/referral; COVID-19: coronavirus disease 2019

Most doctors (n=167, 76.7%) advised testing at government-recognized COVID-19 centers (P<0.05). The RT-PCR (89.5%) was considered the gold standard of COVID-19 diagnosis test while the rapid antigen test (RAT) (76.5%) was used for immediate screening and isolation of suspects with contact history. The positive patients and those who had symptoms but negative COVID-19 reports were followed by high-resolution computed tomography (HRCT) (52%) and C-reactive protein (CRP) testing (45%) to ensure that no COVID-19 infection was missed. On applying the statistical tests, it was found that more medical practitioners preferred the RT-PCR test compared to RAT (Chi-square value=12.6; p-value=0.0004) (Figure 5).

**Figure 6:**
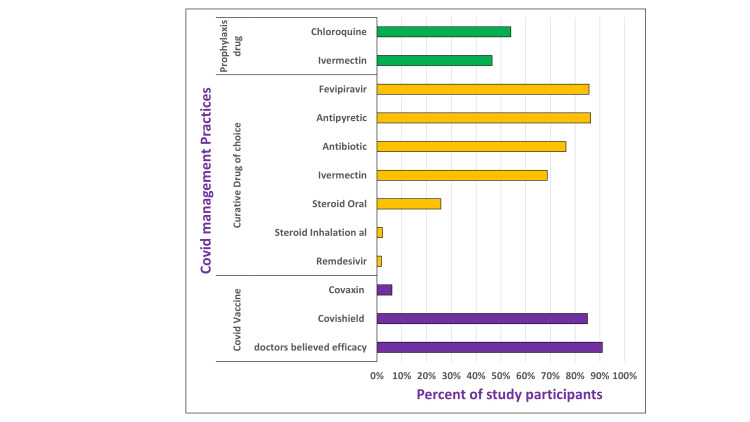
Medical practitioners’ attitudes and practices with regard to drugs and vaccines for COVID-19 management Prophylactic drug was advised to patients having history of contact and negative report. COVID-19: coronavirus disease 2019

The most common drug type used for COVID-19 patients was antipyretic (86.3%) followed by favipiravir (85.2%), antibiotic (76.4%), ivermectin (anti-parasitic) (68.7%), oral steroid (25.8%), remdesivir (anti-viral) (6.6%). Chloroquine (54%) was the most preferable prophylactic drug followed by ivermectin (49.2%) (Fig 6). The majority, 90%, believed that any type of vaccination against COVID-19 infection can prevent morbidity and mortality. If given a choice, Covishield was the preferred vaccine of choice over Covaxin for every group population.

Concerns faced by medical practitioners during the COVID-19 pandemic

Among 191 respondents, n=180 (94.2%) faced personal problems both during and after lockdown in the COVID-19 era. During the COVID-19 pandemic, 7% of doctors (n=14) had to temporarily suspend their practices during lockdown due to physiological reasons like age above 60 years, pregnancy, or co-existing morbidities such as diabetes and hypertension. Increased reporting, Isolation of cases and contacts, triage and management of flu cases, and social distancing were considered by the majority of doctors (range 62-93%) to prevent an explosive occurrence of cases in COVID-19 (Table 3).

**Table 3:** Concerns and measures of private and public sector doctors for safety during the COVID-19 pandemic COVID-19: coronavirus disease 2019

Main concerns (n=191)	Influencing factors	Proportion (%)
Closed clinic/hospital	No	93
	Yes, only during lockdown	7
Personal problems and emotional stress	Elderly parents or children	69.1
	Anxiety or sickness	71.7
	Staff and peers	47.1
	Government policies or logistics	71.7
Practices to reduce disease	Increase reporting	63.7
	Social distancing	96.3
	Flu management	62.3
	Triage	61.4
	Isolation	72.3
	Lockdown	14.1

**Figure 7:**
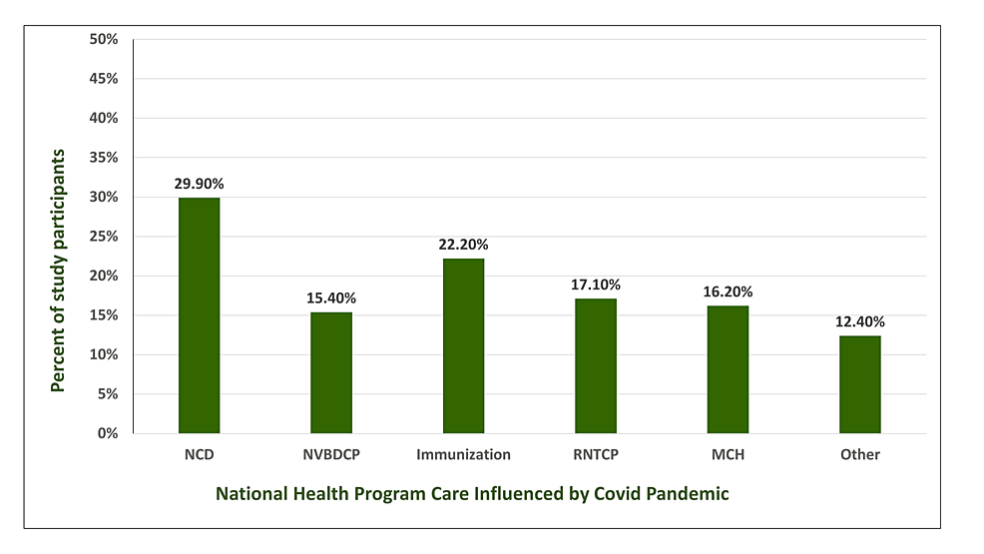
Gaps in government healthcare program delivery during COVID-19 pandemic This simple column chart shows the proportion of doctors who saw that covid-19 pandemic influenced the healthcare delivery for problems. NVBDCP: National Vector-borne disease Control Program; NCD: National Non-Communicable Disease prevention and control program; RNTCP: Revised National Tuberculosis Control Program; MCH: Mother and Child Healthcare program; COVID-19: coronavirus disease 2019

The COVID-19 response by the healthcare system affected the national health programs according to 47% or one-half of healthcare providers (Figure 7). Approximately 90% of public and 10% of private doctors expressed that long-term care components for chronic lifestyle and communicable diseases like hypertension and tuberculosis respectively were discontinued due to fear of transmission of COVID-19 and overworked medical and para-medical persons.

## Discussion

The COVID-19 pandemic brought severe challenges to healthcare providers. At the same time, it was an opportunity to develop better practices and to increase infection transmission awareness among doctors as well as the community [13]. To the best of our knowledge, this is one of the few studies involving a large sample size of qualified doctors including both private and public sector doctors who reported/referred cases of COVID-19. The ratio of private sector doctors and public sector doctors in this study (70:30) was similar to the Indian population’s health-seeking behavior [9]. Both young and old doctors from various areas of specialty having diverse practices were included to get a holistic picture (Figures 2-3).

Healthcare utilization and patient consultation

After the pandemic, patients’ consultation frequencies decreased at the clinics and a significant increase was seen in the virtual consultation methods. This served as an evolving telemedicine model [14] to provide contactless treatment [8,9]. However, it's important to note that the correlation between patient consultations before and during the pandemic suggests a certain level of consistency in patient care, despite the challenges posed by the pandemic (r-value: 0.6-0.78).

Infection prevention practices

Standard precautions were the commonly followed method for preventing transmission of infection, as per established guidelines for infection control. Social distancing, reducing the number of patients, and consultation hours reported a statistically significant increase (P<0.001), indicating that healthcare practitioners' behavior changed to minimize exposure as they tried to safeguard against infection [5].

COVID-19 diagnosis and management

The preference for RT-PCR over RAT for COVID-19 diagnosis in this study reflects the higher sensitivity, reliability, and accuracy of the former method. Furthermore, government-recognized laboratories and tests were more trusted even by private doctors showing public-private collaboration during situation of covid pandemic in this study. The negligible cost of testing in public setups and standard testing procedures like RT-PCR followed by HRCT scans [2] was the standard procedure for comprehensive diagnosis and monitoring of COVID-19 cases (Figure 5).

This study tried to explore the established treatment protocol by city practitioners in Surat. In terms of drug usage, antipyretics, favipiravir, antibiotics, and ivermectin were commonly prescribed (Figure 6). These findings are in line with evolving treatment protocols during the pandemic, with antipyretics providing symptom relief and antiviral drugs like favipiravir being explored for their potential benefits [2]. However, the use of antibiotics in COVID-19 treatment should be approached with caution to avoid contributing to antibiotic resistance. For prophylaxis, ivermectin and chloroquine were the preferred medicines in this study. It was worth noting that the National Task Force for COVID-19 recommended the use of hydroxychloroquine drug for prophylactic use in selected individuals [15]. Nevertheless, a review of the literature advises caution against the development of false security, more observational research, and clinical trials before providing chloroquine or hydroxychloroquine to asymptomatic individuals [16].

Vaccination and concerns

The study reported a positive attitude towards COVID-19 vaccination among medical practitioners. This aligns with the global effort to promote vaccination as a primary preventive measure against COVID-19 [17]. The preference for Covishield over Covaxin suggests the influence of factors such as vaccine availability, public perception, and clinical trial data on vaccine preference.

Health practitioners suffered a great challenge both financially and mentally, especially during the COVID-19 lockdown [8]. Personal concerns, stress, and disruptions in healthcare services were evident among both private and public sector doctors (Table 3). These concerns were driven by various factors including age, health conditions, logistical challenges, and government policies. The impact of the pandemic on healthcare delivery was substantial, as evidenced by the discontinuation of long-term care components for chronic diseases.

According to the doctors, routine work of national health programs and care delivery for non-communicable diseases management (n=70; 30%), immunization (n=52; 22%), tuberculosis (n=40; 17%), and vector-borne disease control (n=26; 12%) had to bear the maximum brunt as these diseases require long-term care and frequent follow-up visits of health centers. Mother and Child healthcare programs also suffered a setback (n=38; 17%) in reaching the beneficiaries during the pandemic.

Limitations and future implications

While this study provides valuable insights in several important aspects of medical response and healthcare delivery during the COVID-19 global health crisis among a large sample size of both private and public sector doctors, there are some limitations to consider. The diversity of healthcare practitioners' specialties, fields, areas, and active involvement in COVID-19 case management was taken into account during recruitment and sample selection, which was an overall strength but we could not study indigenous, non-traditional medicine practitioners or practitioners of alternative treatments like yoga, meditation, and healing practices prevalent during COVID-19 pandemic [18]. Additionally, self-reporting and recall bias might have influenced the responses. Furthermore, the study did not delve deeply into the patients’ outcomes of the evolving medical practices established in this study. This along with reasons behind certain practices, attitudes, and concerns, could be explored in future research.

The findings underscore the need for well-coordinated responses from both public and private healthcare sectors during a pandemic. Future strategies should focus on addressing the concerns and stressors faced by healthcare practitioners, ensuring the continuation of essential health programs, and maintaining a balanced approach to patient care amid evolving pandemic dynamics.

## Conclusions

This study provides a comprehensive overview of preparedness, evolving patterns, diagnosis, management practices, and concerns of medical practitioners in an urban area of western India during the COVID-19 pandemic. The insights gained from this research can inform policy decisions and healthcare strategies to better equip healthcare systems to handle similar crises in the future and to ensure the well-being of both healthcare providers and patients during global health emergencies.
